# Bi-articular Knee-Ankle-Foot Exoskeleton Produces Higher Metabolic Cost Reduction than Weight-Matched Mono-articular Exoskeleton

**DOI:** 10.3389/fnins.2018.00069

**Published:** 2018-03-02

**Authors:** Philippe Malcolm, Samuel Galle, Wim Derave, Dirk De Clercq

**Affiliations:** ^1^Department of Biomechanics, Center for Research in Human Movement Variability, University of Nebraska Omaha, Omaha, NE, United States; ^2^Department of Movement and Sports Sciences, Ghent University, Ghent, Belgium

**Keywords:** bi-articular, mono-articular, exoskeleton, walking, gastrocnemius, soleus, metabolic cost, pneumatic muscles

## Abstract

The bi-articular m. gastrocnemius and the mono-articular m. soleus have different and complementary functions during walking. Several groups are starting to use these biological functions as inspiration to design prostheses with bi-articular actuation components to replace the function of the m. gastrocnemius. Simulation studies indicate that a bi-articular configuration and spring that mimic the m. gastrocnemius could be beneficial for orthoses or exoskeletons. Our aim was to test the effect of a bi-articular and spring configuration that mimics the m. gastrocnemius and compare this to a no-spring and mono-articular configuration. We tested nine participants during walking with knee-ankle-foot exoskeletons with dorsally mounted pneumatic muscle actuators. In the *bi-articular plus spring condition* the pneumatic muscles were attached to the thigh segment with an elastic cord. In the *bi-articular no-spring condition* the pneumatic muscles were also attached to the thigh segment but with a non-elastic cord. In the *mono-articular* condition the pneumatic muscles were attached to the shank segment. We found the highest reduction in metabolic cost of 13% compared to walking with the exoskeleton *powered-off* in the *bi-articular plus spring condition*. Possible explanations for this could be that the exoskeleton delivered the highest total positive work in this condition at the ankle and the knee and provided more assistance during the isometric phase of the biological plantarflexors. As expected we found that the *bi-articular conditions* reduced m. gastrocnemius EMG more than the *mono-articular condition* but this difference was not significant. We did not find that the *mono-articular condition* reduces the m. soleus EMG more than the *bi-articular conditions*. Knowledge of specific effects of different exoskeleton configurations on metabolic cost and muscle activation could be useful for providing customized assistance for specific gait impairments.

## Introduction

In most mainstream human-like robots (e.g., ASIMO, Sakagami et al., 2002), each degree of freedom of every joint is controlled by a separate actuator (Collins et al., 2005). Humans have not only muscles that actuate one joint but also muscles that cross two joints. These so-called bi-articular muscles, such as the m. gastrocnemius and biceps femoris, at first seem to be an unnecessarily complicated evolutionary adaptation for actions that could in principle be accomplished by mono-articular muscles. However, multiple sources of evidence point toward the benefits of biological bi-articular muscles (van Ingen Schenau, 1990). Bi-articular muscles facilitate the coupling of joint movements and allow to control distal joints via tendons connected to proximally located muscles, thereby reducing distal mass (Cleland, 1867). They also transport work from proximal mono-articular muscles to distal joints (Elftman, 1939; Van Ingen Schenau et al., 1987) while requiring lower shortening velocities from these muscles (Cleland, 1867; Bobbert and van Ingen Schenau, 1988). In walking, the bi-articular m. gastrocnemius has functions that differ from but are complementary to the functions of the mono-articular m. soleus (Neptune et al., 2001; Gottschall and Kram, 2005; Sasaki and Neptune, 2006; McGowan et al., 2008).

Several authors have proposed to use biological bi-articular muscles as inspiration in the design of robotic prostheses and exoskeletons (Ferris et al., 2007; Junius et al., 2017). Different groups are developing ankle prostheses with bi-articular components (Endo et al., 2009; Grimmer and Seyfarth, 2009; Eslamy et al., 2015; Flynn et al., 2015; Willson et al., 2015; Eilenberg, 2017) intended to mimic the function of the biological m. gastrocnemius. This gastrocnemius muscle has its origin on the medial and lateral epicondyles of the femur, inserts onto the calcaneus and performs primarily plantarflexion and secondary knee flexion. A simulation study (Eslamy et al., 2015) indicated that the addition of a gastrocnemius-mimicking bi-articular component could reduce the motor energy requirements of robotic prostheses. With respect to assistive devices that work in parallel with the body, different groups are designing exoskeletons and exosuits with multi-articular couplings (Dean, 2009; Bartenbach et al., 2015), often with non-biologically inspired configurations such as coupling plantarflexion with hip flexion (van den Bogert, 2003; van Dijk et al., 2011; Asbeck et al., 2013).

In contrast to studies in the field of prostheses, to our knowledge, no group has experimentally evaluated a configuration that mimics the biological m. gastrocnemius in exoskeletons. Exoskeletons are defined as anthropomorphic wearable devices that fit closely to the body and work in concert with the operators movements (Herr, 2009) and can be used for applications such as gait rehabilitation or assistance in clinical populations and augmentation in healthy populations. Exoskeletons with separate knee and ankle actuation have been designed (Sawicki and Ferris, 2009; Chen et al., 2016). However, a coupling similar to the m. gastrocnemius has only been evaluated in simulation.

A musculoskeletal simulation study by Arch et al. indicated that (mono-articular) ankle-foot orthoses do not sufficiently replicate the function of the m. gastrocnemius (Arch et al., 2016). Another simulation study by Baskar and Nadaradjane indicated that a bi-articular spring could potentially reduce the metabolic rate (Baskar and Nadaradjane, 2016). In their simulation they found that this bi-articular spring reduced the metabolic rate of the m. gastrocnemius, m. soleus and m. iliopsoas. It is uncertain to what extent the aforementioned simulation predictions would translate into experimental results. Previous simulation studies of exoskeletons (Farris et al., 2014; Van Dijk, 2015; Sawicki and Khan, 2016) often do not provide exact predictions of experimental results (van Dijk et al., 2011; Farris and Sawicki, 2012; Collins et al., 2015), which is likely due to the difficulty of predicting how a wearer will interact with an assistive exoskeleton (Gordon et al., 2006).

Our aim was to experimentally test the physiological and biomechanical effects of bi-articular configurations that mimic the biological m. gastrocnemius in healthy participants. To understand the specific effects of a bi-articular actuation path (based on the presence of bi-articular muscles in humans) and a bi-articular spring (based on the study by Baskar and Nadaradjane, 2016), we compared multiple configurations with bi-articular and mono-articular configurations either with or without a spring. We hypothesized that a mono-articular soleus-mimicking configuration would lead to a higher reduction in m. soleus EMG. We also hypothesized that bi-articular gastrocnemius-mimicking configurations would lead to higher reductions in m. gastrocnemius EMG. Finally, we hypothesized that bi-articular gastrocnemius-mimicking conditions would lead to higher reductions in metabolic rate compared to mono-articular conditions because they would provide additional assistance at the knee.

## Materials and methods

### Participants

**Figure 1:**
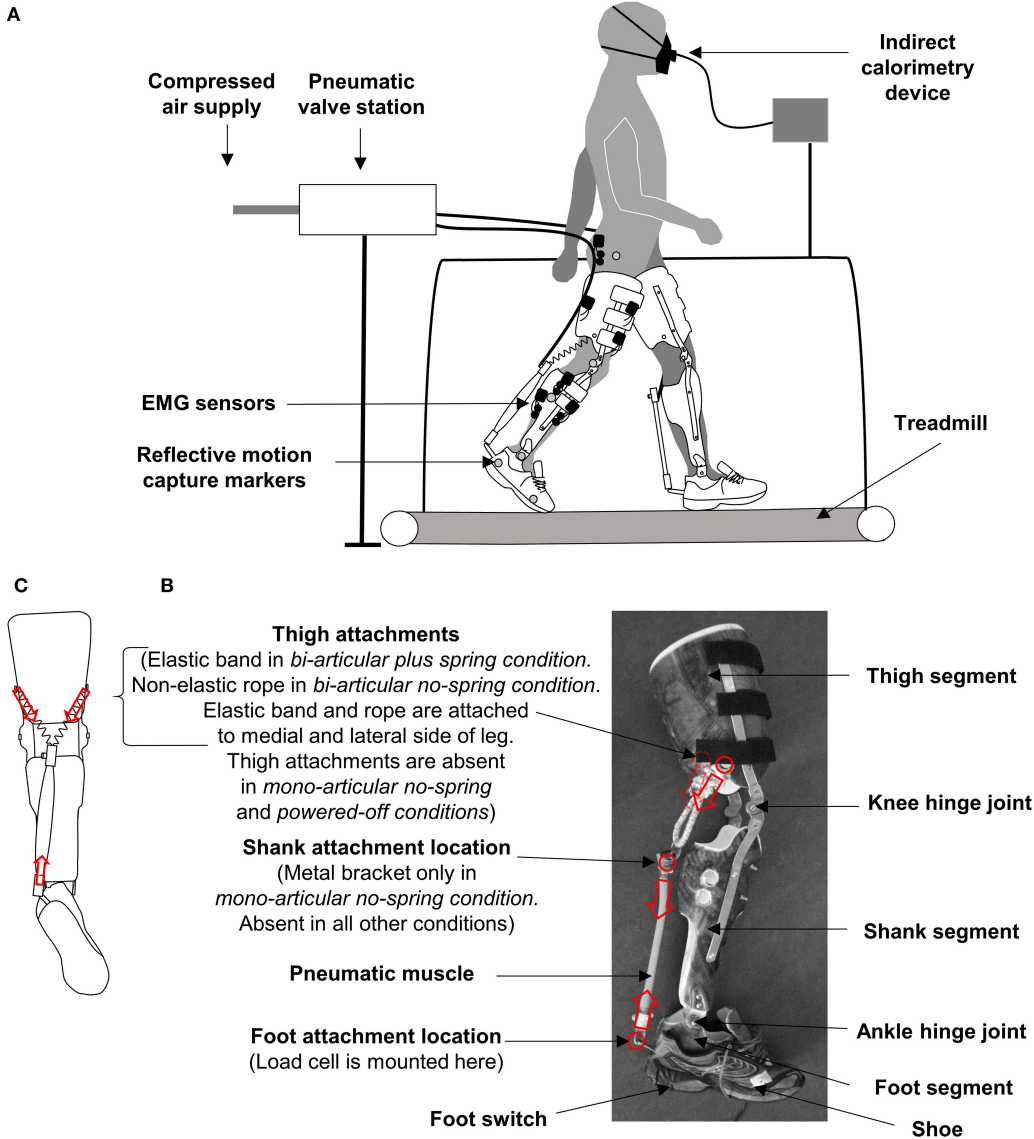
Methods. **(A)** Experimental setup. **(B)** Exoskeleton. Red circles and arrows indicate attachment locations and pulling force directions. **(C)** Schematic rear view of exoskeleton in the *bi-articular conditions* showing the origin of the pneumatic muscle attachment on the medial and lateral side of the thigh segment.

We tested nine healthy participants (7♂, 2♀, 71 ± 2 kg, 177 ± 1 cm, 23 ± 1 year, values are mean ± standard error) during walking at 1.25 ms^−1^ on a treadmill (Figure 1). Since we did not have a prior estimate for the effect size for different exoskeleton configurations the number of participants was chosen based on other exoskeleton studies that demonstrate significant within-participant effects of different actuation types with 7–10 participants (Sawicki and Ferris, 2008; Malcolm et al., 2013; Collins et al., 2015; Mooney and Herr, 2016; Galle et al., 2017; Quinlivan et al., 2017). The walking speed of 1.25 ms^−1^ was selected to reflect the preferred walking speed of healthy adults (Rose et al., 1994) and to be similar to the speed that is used in most exoskeleton studies with healthy participants (Sawicki and Ferris, 2008; Malcolm et al., 2013; Collins et al., 2015; Galle et al., 2017). All participants of the study provided written informed consent prior to participation. The ethics committee of the Ghent University Hospital approved the protocol (Belgian registration number B670220097074).

### Exoskeleton

The participants wore bilateral hinged knee-ankle-foot exoskeletons powered by pneumatic artificial muscles (Figure 1, Movie 1). The exoskeleton consisted of three shells that fit around the foot, lower leg, and thigh. The shells were molded with thermoplastic on a person with average morphology. The shells were connected with orthopedic steel bars and hinge joints. The steel bars were situated both on the medial and lateral side of the leg.

The height of the hinge joint for the knee joint was adjusted to match the participants' anthropometry. The exoskeleton was attached to the wearer by means of Velcro straps and tape around the thigh and lower leg segment. The lower leg segment of the exoskeleton weighed 0.65 kg per side, and the thigh segment of the exoskeleton weighed 1.25 kg per side. The design of our new bi-articular exoskeleton was based on our previous ankle exoskeleton (Malcolm et al., 2013) with the addition of a thigh segment. The final design of the anchor points was based on a series of pilot tests and design modifications. The exoskeleton was tethered to a stationary power source and control unit. This type of tethered setup is similar to other knee-ankle-foot exoskeletons intended for biomechanics studies (Sawicki and Ferris, 2009) but it does not allow overground locomotion in contrast to certain other knee-ankle-foot exoskeletons (Chen et al., 2016).

### Actuation control

We actuated the exoskeletons with pneumatic artificial muscles of 3 cm in diameter. A computer program (LabView, National Instruments, Austin, TX, USA) was used to trigger the onset and end of the pneumatic muscle contraction at different percentages of the stride cycle based on signals from heel switches (Mec, Ballerup, Denmark). The pneumatic muscles were made to contract and lengthen by opening and closing of pneumatic valves (Festo, Esslingen, Germany). A constant supply pressure of 3.5 bar was used. The exact behavior of the pneumatic muscles was dependent on the inflation and deflation of the pneumatic muscles, the force-pressure-length relationship and the kinematics and the kinetics of the participant. This actuation control system was similar to the system used in Malcolm et al. (2013).

### Conditions

**Figure 2:**
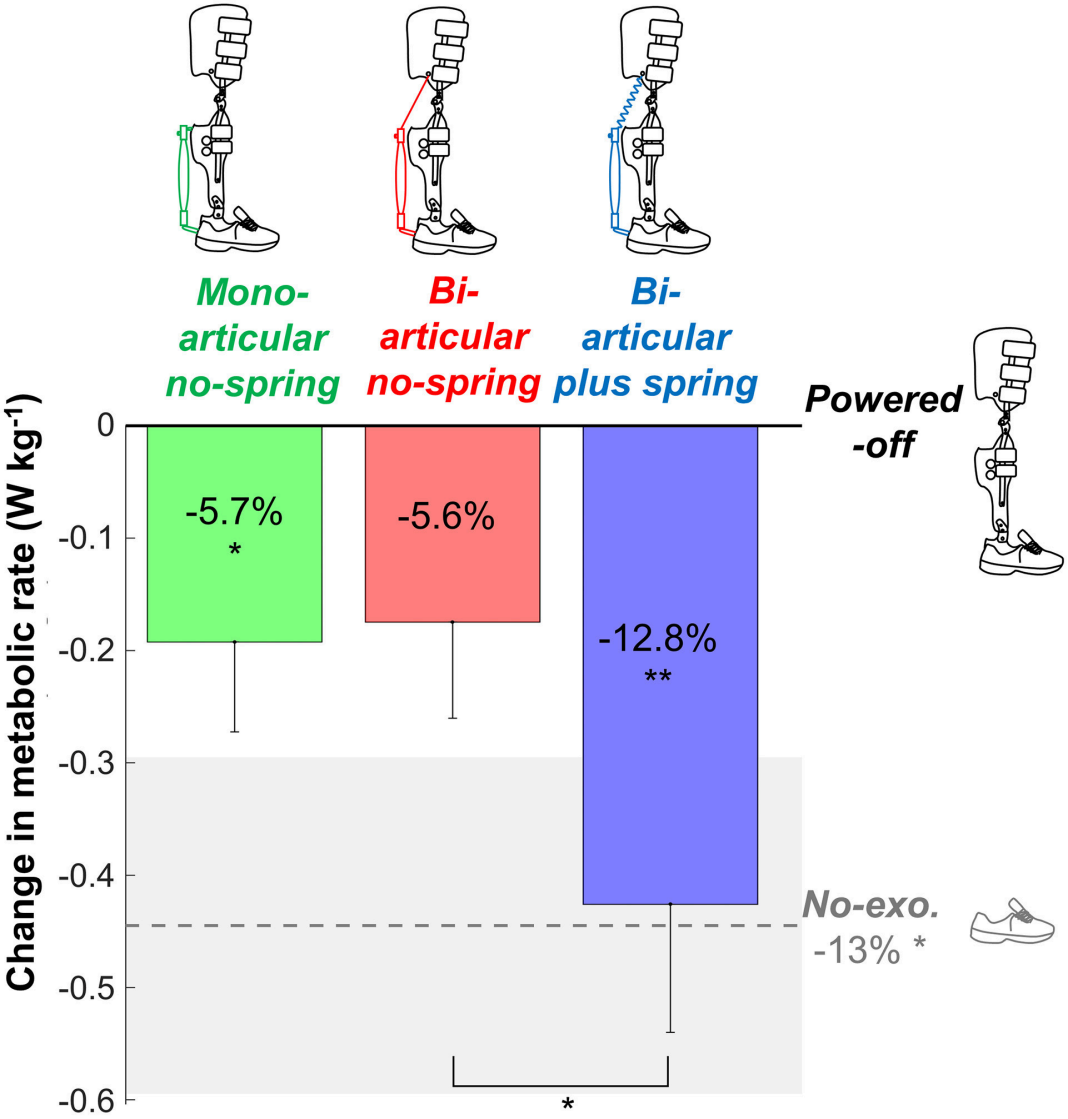
Metabolic rate. Vertical bars show the change in metabolic rate compared to walking while wearing the exoskeleton *powered-off*. Differently colored bars represent the different exoskeleton configurations (shown in pictograms). The dashed gray line represents the difference in metabolic rate between the *no-exoskeleton* and *powered-off* conditions. Error bars indicate standard error. Shaded area indicates standard error for the *no-exoskeleton* condition. Percentages indicate percent reduction. Symbols in the vertical bars indicate significant differences compared to *powered-off*. Symbols below brackets indicate significant differences between other exoskeleton configurations. ^**^p ≤ 0.01, ^*^p ≤ 0.05.

We tested five conditions (Figure 2, Movie 1):

In the *bi-articular plus spring condition*, we attached the proximal end of the pneumatic muscles to the medial and lateral sides of the thigh segment via an elastic cord and a second non-elastic cord in parallel with the elastic cord that served to limit the maximum elongation. When pilot testing the *bi-articular plus spring condition*, we determined based on subjective perception that this second non-elastic rope was necessary to achieve an assistive effect during push-off because pneumatic muscles can only contract up to about 30% of their resting length. We intended this configuration to assist both during the eccentric and the concentric phase of the m. gastrocnemius contraction.

In the *bi-articular no-spring condition*, we attached the proximal end of the pneumatic muscles to the medial and lateral side of the thigh segment only via a non-elastic elastic cord. We intended this configuration to assist mostly with the concentric phase of the m. gastrocnemius contraction.

In the *mono-articular no-spring condition*, we attached the proximal end of the pneumatic muscles to the shank. This attachment configuration was roughly similar to that used in our previous study (Malcolm et al., 2013) except that the participants wore the non-functional thigh segment of the exoskeleton to prevent the exoskeleton mass difference from confounding the effects of differences in the actuation configuration. We did not have a mono-articular plus spring condition because we were not able to fit a pneumatic muscle that would contract over a sufficient distance and a spring within the length of the shank segment of the exoskeleton.

In the *powered-off condition*, the participants wore the entire exoskeleton without actuators.

In the *no-exoskeleton condition*, the participants walked with normal shoes without the exoskeleton.

In all *active conditions* (i.e., the *bi-articular plus spring, bi-articular no-spring*, and *mono-articular no-spring condition*) we positioned the pneumatic muscles such that they had a moment arm of ~11 cm vs. the ankle joint. In the *bi-articular condition*s, the proximal attachment was positioned on the thigh such that in the standing position, the moment arm vs. the knee joint was about half of the moment arm vs. the ankle in order to mimic the biological moment arm ratio of the m. gastrocnemius (Hof, 2001). In all three *active conditions*, we mounted the pneumatic muscles such that they appeared to be maximally elongated with the knee fully extended and ankle in 15° dorsiflexion. In the *bi-articular plus spring condition*, the elastic cord was tensioned such that it starts providing force with the knee fully extended and the ankle in 5° plantarflexion. Both adjustments were performed using tensioning screws on the pneumatic muscles while the participant stood on a 15 or 5° slope. Both angles were selected during pilot test in order to achieve that in the *bi-articular plus spring condition* the pneumatic muscles would be maximally elongated and start to apply force at initial forefoot contact (i.e. the beginning of the eccentric phase of the plantarflexors) and the *bi-articular no-spring* and *mono-articular condition* would reach maximum elongation at the beginning of the concentric phase of the plantarflexors.

### Protocol

Before the metabolic and biomechanical testing protocol, the participants were allowed 18 min (Galle et al., 2013) of habituation to the different exoskeleton configurations with different timings. We used a perception-based optimization method (Caputo, 2015) to determine the optimal onset timing control input in each *active condition*. In one trial, we gradually shifted the onset timing from 23 to 53% of the stride until the participant verbally indicated that the actuation onset timing went past their perceived optimum. In another trial, we followed the same procedure in the opposite direction. We then used the mean values from the ascending and descending trials as control inputs for further metabolic and biomechanical testing. For the actuation ending, we always used a fixed control input of 60% of the stride. In the metabolic and biomechanical testing protocols, participants walked 4 min in each condition and rested while we changed the configurations. We randomized the order of the conditions.

### Measurements

We recorded respiratory O_2_ consumption and CO_2_ production via indirect calorimetry (Oxycon Pro, Jaeger GMBH, Höchberg, Germany). We recorded the force from the pneumatic muscles at a rate of 1,000 fps with a load cell (210 Series, Richmond Industries Ltd., Rearing, United Kingdom) mounted on the distal end of the pneumatic artificial muscles such that the load cell registered the total force from the pneumatic muscle (and the series elastic cord and/or non-elastic cord in the *bi-articular conditions*). The load cell data from one participant are missing due to a device malfunction. We recorded the muscle activation on the right leg of the m. tibialis anterior, soleus, gastrocnemius medialis, gastrocnemius lateralis, vastus lateralis, rectus femoris, biceps femoris, and gluteus maximus at a rate of 1,000 fps using surface EMG sensors (Noraxon, Scottsdale, AZ, USA). We measured the kinematics of the right leg using sagittal video recording at a rate of 200 fps (Basler AG, Ahrensburg, Germany) and reflective markers on the forefoot, ankle, knee and trochanter. We recorded heel contact times using the foot switches of the exoskeleton. We processed indirect calorimetry measurements for the last 2 min of each 4-min condition. We collected load cell, EMG, kinematic, and temporal data only during the *exoskeleton conditions* (i.e., the *active conditions* and the *powered-off condition*) over a 10-second period during the last minute of each condition.

### Data processing

We calculated the metabolic rate using the Brockway equation (Brockway, 1987) and a measurement of the resting metabolic rate while standing to obtain the net metabolic rate for the walking conditions. We rectified the EMG data, applied a band pass filter (50–450 Hz) and then calculated a moving root mean square with a window of 100 ms. We normalized the EMG data to the average peak value per stride in the *powered-off* condition. We filtered the marker data with a 12-Hz Butterworth lowpass filter. Based on visual inspection, we excluded the EMG data from 24 trials (out of 288 in total) due to the presence of artifacts. We calculated the sagittal plane joint angles and angular velocities of the ankle, knee, and hip joints. For each joint angle, we subtracted the joint angle in the standing position. We estimated the total exoskeleton ankle moment by multiplying the load cell force by the moment arm vs. the ankle. In the *bi-articular condition*, we estimated the exoskeleton knee moment by multiplying the load cell force by the moment arm vs. the knee. We calculated the total exoskeleton power vs. the ankle and the knee by multiplying the exoskeleton moments by the joint angular velocities, and we calculated the positive exoskeleton work rates by integrating the positive portions of the exoskeleton power over time and dividing by the stride time. We calculated the step length by multiplying the step times obtained from foot switches by the speed of the treadmill. All time-series data were normalized vs. the stride time based on heel contact detection by the foot switches. We calculated the minima and maxima from all normalized stride time data, and we calculated the onset timing from the exoskeleton ankle moment data.

### Statistics

For each time series and metric, we calculated the mean and standard error. For each metric, we tested whether there were any effects of exoskeleton condition using repeated measures ANOVA. We used Mauchly's test to verify sphericity and used the Greenhouse-Geiser correction if the sphericity assumption was violated. If the repeated measures ANOVA indicated a significant effect, we evaluated pairwise differences using paired *t*-tests using the least significant difference method. For the actuation onset timing metric, we tested whether there was a significant difference between the value in the *mono-articular no-spring condition* and the value of 42.5%, which was the average optimal timing of earlier publications with a similar mono-articular actuator configuration (Malcolm et al., 2013; Galle et al., 2017) using a one-sample *t*-test. We also tested whether the exoskeleton work rate had a significant linear effect on the reduction of the metabolic cost using mixed-model ANOVA. For the repeated measures ANOVA we reported the degrees of freedom of the condition, the degrees of freedom of the error, the *f*-value, the *p*-value, and the partial eta squared. For the *t*-tests we reported the degrees of freedom, the *t*-value, the *p*-value, and the Cohen's *d*. For the mixed-model ANOVA we reported the degrees of freedom, the *t*-value, the *p*-value, and the *R*^2^ between the estimated metabolic cost (obtained using the equation obtained from the mixed-model ANOVA) and the actual metabolic cost. All statistical tests were conducted in MATLAB (MathWorks, Natick, MA, USA).

## Results

### Exoskeleton mechanics

**Figure 3:**
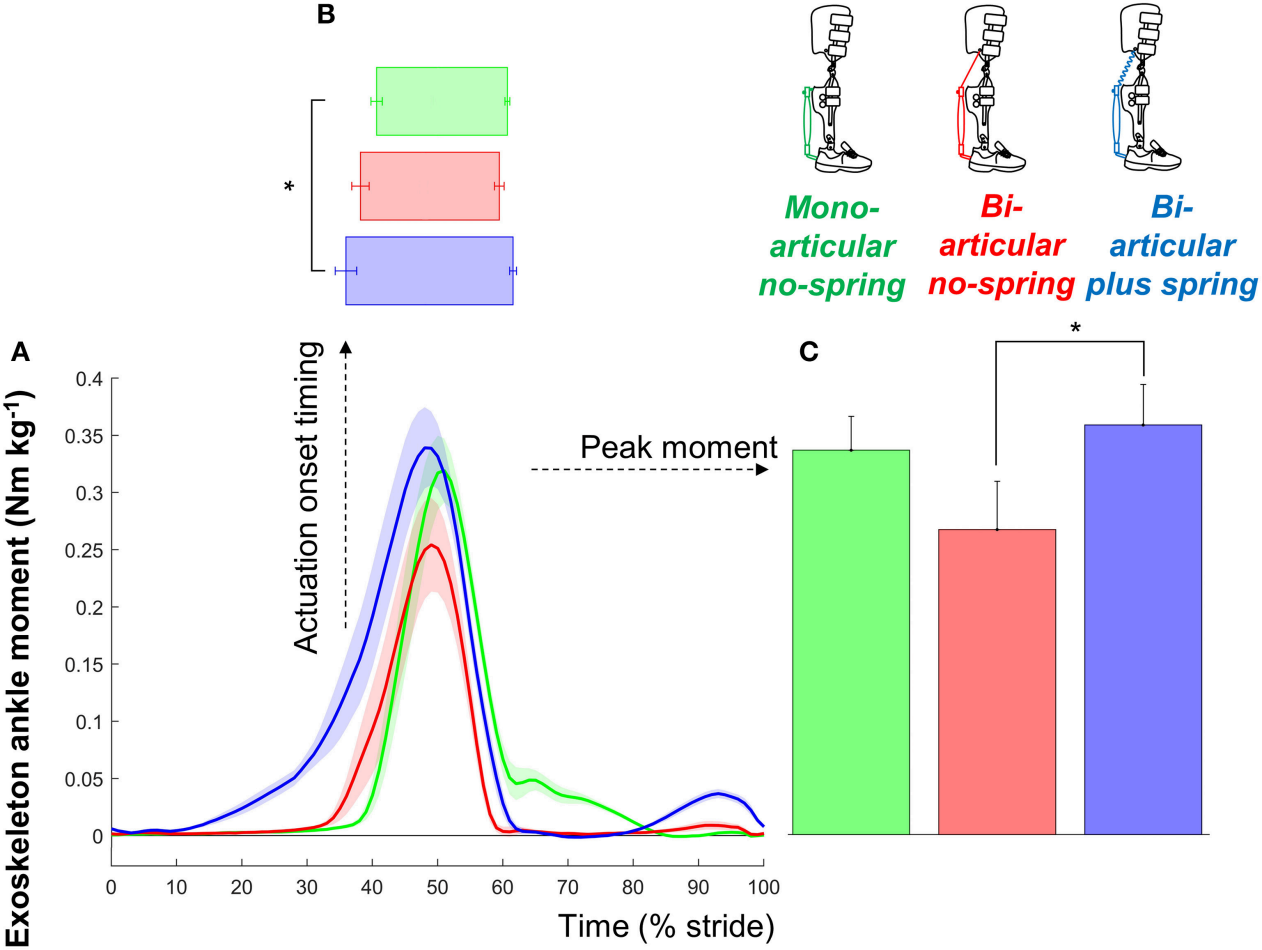
Exoskeleton moment. **(A)** Exoskeleton ankle moment vs. stride time. Colored lines indicate the population average of different conditions (shown in pictograms). Shaded areas indicate standard error. **(B)** Actuation onset timing. **(C)** Peak moment. Differently colored bars represent the different exoskeleton configurations. Error bars indicate standard error. Symbols next to brackets indicate significant differences between exoskeleton configurations. ^*^p ≤ 0.05.

Participants selected actuation onsets of 36.1 ± 1.6, 38.4 ± 1.3, and 40.9 ± 0.9% in the *bi-articular plus spring condition, bi-articular no-spring condition*, and *mono-articular no-spring condition*, respectively (Figure 3). Exoskeleton configuration had a significant effect on the onset timing obtained from the perception optimization (*df*_cond._ = 2, *df*_err._ = 14, *F* = 4.080, *p* = 0.040, η^2^ = 0.368). The selected actuation onset was significantly earlier in the *bi-articular plus spring condition* than in the *mono-articular no-spring condition* (*df* = 7, *T* = 2.927, *p* = 0.022, *d* = 1.034). The selected actuation onset in the *mono-articular no-spring condition* was not significantly different from the average value from previous publications in which similar mono-articular soleus-mimicking configurations were used (Malcolm et al., 2013; Galle et al., 2017) (*df* = 7, *T* = 1.739, *p* = 0.126, *d* = 0.61, one-sample *t*-test vs. 42.5%).

The peak exoskeleton ankle moments were 0.35 ± 0.04, 0.26 ± 0.04, and 0.33 ± 0.03 Nm kg^−1^ in the *bi-articular plus spring condition, bi-articular no-spring condition*, and *mono-articular no-spring condition*, respectively. Exoskeleton configuration had a significant effect on the peak exoskeleton moment (*df*_cond._ = 2, *df*_err._ = 14, *F* = 5.503, *p* = 0.017, η^2^ = 0.440). The peak exoskeleton ankle moment in the *bi-articular plus spring condition* was higher than that in the *bi-articular no-spring condition* (*df* = 7, *T* = 2.934, *p* = 0.022, *d* = 1.037). The elastic cord in the *bi-articular plus* spring condition resulted in an average angular stiffness of 2.662 Nm°^−1^ around the ankle during the phase before the pneumatic muscle contraction.

The peak exoskeleton positive ankle work rates were 0.065 ± 0.006, 0.044 ± 0.006, 0.103 ± 0.011 W kg^−1^ per side in the *bi-articular plus spring condition, bi-articular no-spring condition*, and *mono-articular no-spring condition*, respectively. The peak exoskeleton positive knee work rates were 0.110 ± 0.011 and 0.070 ± 0.013 W kg^−1^ per side in the *bi-articular plus spring condition* and *bi-articular no-spring condition*, respectively.

### Metabolic rate

Net metabolic rates were 2.79 ± 0.12 W kg^−1^ in the *bi-articular plus spring condition*, 3.04 ± 0.15 W kg^−1^ in the *bi-articular no-spring condition*, 3.02 ± 0.12 W kg^−1^ in the *mono-articular no-spring condition*, 3.21 ± 0.13 W kg^−1^ in the *powered-off condition*, and 2.77 ± 0.12 W kg^−1^ in the *no-exoskeleton condition* (Figure 2). Exoskeleton configuration had a significant effect (*df*_cond._ = 4, *df*_err._ = 32, *F* = 4.832, *p* = 0.004, η^2^ = 0.377) on the net metabolic rate. The metabolic rate in the *bi-articular plus spring condition* was 12.8 ± 3.1% lower than that in the *powered-off condition* (*df* = 8, *T* = 3.734, *p* = 0.006, *d* = 1.245), and lower than that in the *bi-articular no-spring condition* (*df* = 8, *T* = 2.920, *p* = 0.019, *d* = 0.973). The metabolic rate in the *bi-articular no-spring* condition was on average 5.6 ± 2.7% lower than in the *powered-off* condition but this difference was not significant (*df* = 8, *T* = 2.042, *p* = 0.075, *d* = 0.681). The metabolic rate in the *mono-articular no-spring condition* was 5.7 ± 2.5% lower than in the *powered-off condition* (*df* = 8, *T* = 2.400, *p* = 0.043, *d* = 0.800). The metabolic rate was 13 ± 4% lower in the *no-exoskeleton condition* than in the *powered-off condition* (*df* = 8, *T* = 2.971, *p* = 0.018, *d* = 0.990). None of the *exoskeleton conditions* reduced the metabolic rate below that of the *no-exoskeleton condition*.

**Figure 4:**
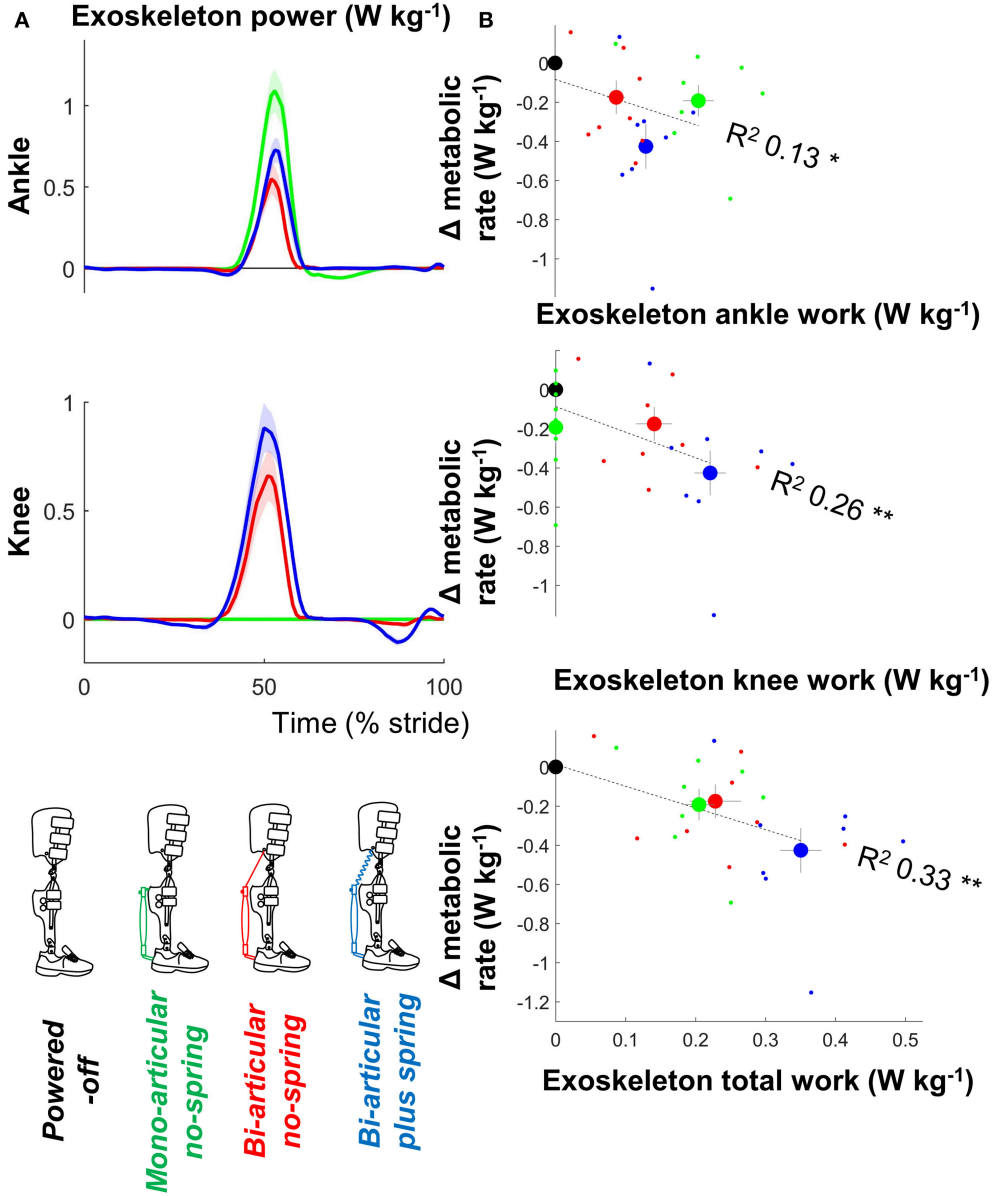
Metabolic rate vs. exoskeleton power and work. **(A)** Exoskeleton ankle and knee power. Since pneumatic muscles can only apply a pulling force, positive ankle power indicates ankle angular velocity in the flexion direction and positive knee power indicates knee angular velocity in the flexion direction. Colored lines indicate the population average of different conditions. Shaded areas indicate standard error. **(B)** Change in metabolic rate vs. positive exoskeleton work. Large dots indicate population average for each condition. Small dots indicate individual trials. Dashed lines indicate results from regression equation from mixed-model ANOVA. R^2^ values are calculated based on Pearson's correlation of estimated results from mixed-model ANOVA vs. actual metabolic results from all participants and conditions. ^**^p ≤ 0.01, ^*^p ≤ 0.05.

There were significant relationships between the metabolic rate and positive ankle work (*df* = 30, *T* = 0.212, *p* = 0.042, *R*^2^ = 0.126, mixed-model ANOVA), positive knee work (*df* = 30, *T* = 3.392, *p* = 0.002, *R*^2^ = 0.260), and positive total joint work (*df* = 30, *T* = 4.175, *p* < 0.001, *R*^2^ = 0.33) (Figure 4).

### EMG

**Figure 5:**
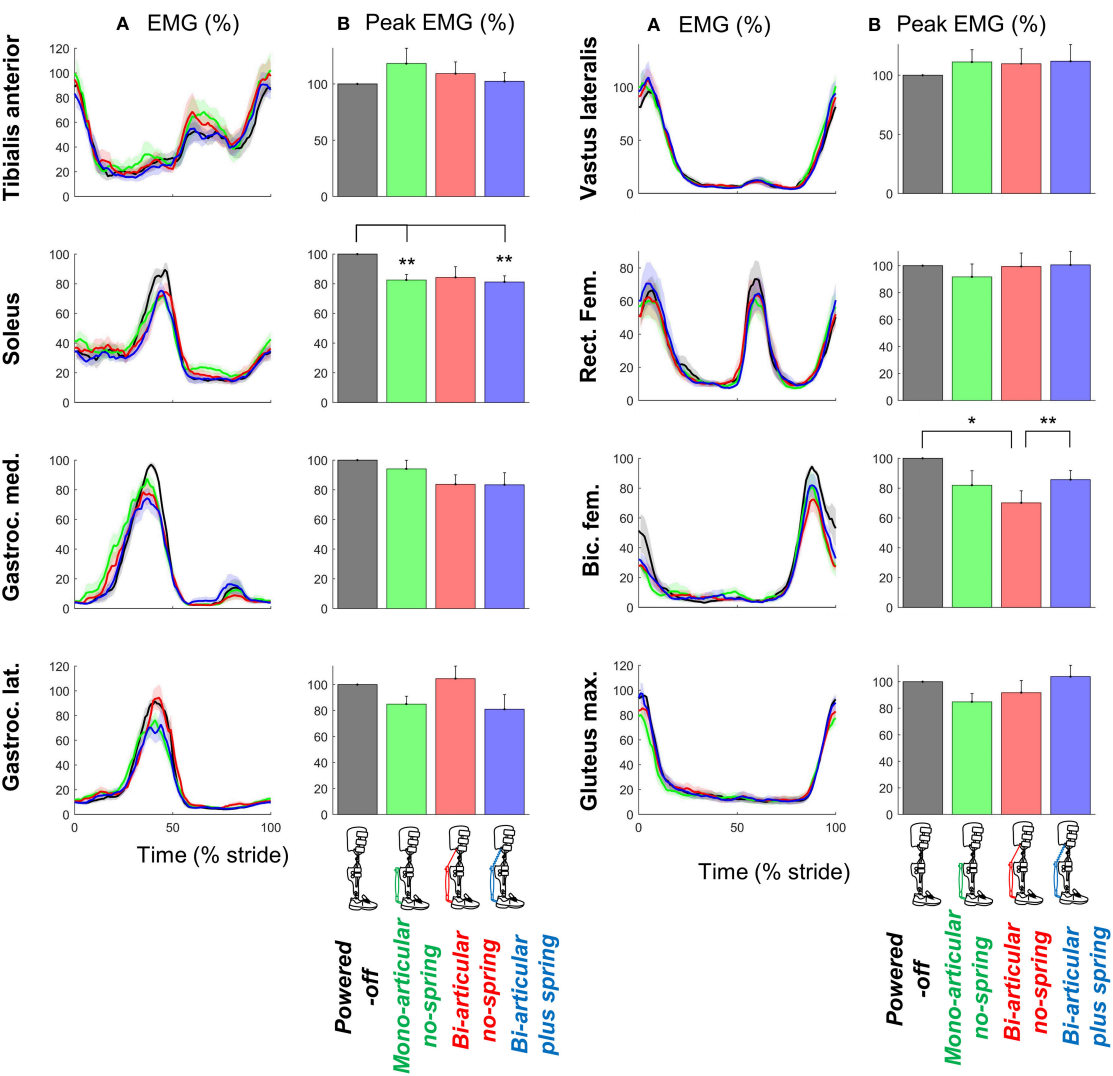
EMG. **(A)** EMG vs. stride time. Different plots are from different muscles. Colored lines indicate the population average of different conditions (shown in pictograms). Shaded areas indicate standard error. **(B)** Peak EMG values for each muscle and condition. Differently colored bars represent the different exoskeleton configurations. Error bars indicate standard error. Symbols next to brackets indicate significant differences between exoskeleton configurations. ^*^p ≤ 0.05. ^**^p ≤ 0.01.

Exoskeleton configuration had significant effects on the peak EMG-values of the m. soleus (*df*_cond._ = 3, *df*_err._ = 18, *F* = 8.882, *p* < 0.001, η^2^ = 0.597) and biceps femoris (*df*_cond._ = 3, *df*_err._ = 15, *F* = 3.895, *p* = 0.031, η^2^ = 0.438) (Figure 5).

The peak m. soleus EMG-values in the *bi-articular plus spring condition* (*df* = 7, *T* = 4.314, *p* = 0.004, *d* = 1.525) and *mono-articular no-spring condition* (*df* = 6, *T* = 4.031, *p* = 0.007, *d* = 1.524) were lower than in the *powered-off condition*.

The peak m. biceps femoris in the *bi-articular no-spring condition* was also lower than that in *the powered-off condition* (*df* = 5, *T* = 3.033, *p* = 0.029, *d* = 1.238) and that in the *bi-articular plus spring condition* (*df* = 5, *T* = 5.178, *p* = 0.004, *d* = 2.114).

### Kinematics

**Figure 6:**
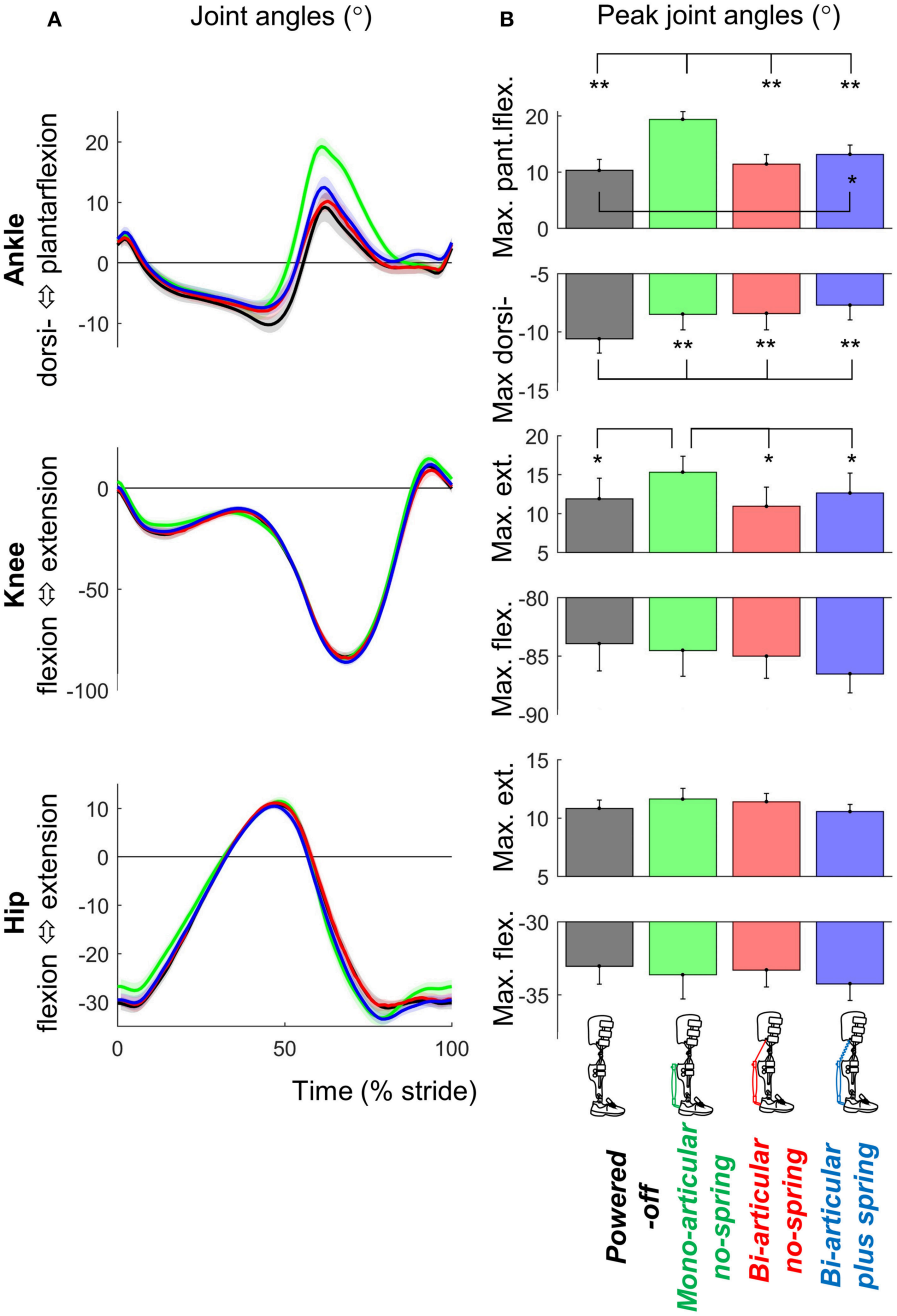
Kinematics. **(A)** Joint angle vs. stride time. Different plots are from different joints. Colored lines indicate the population average of different conditions (shown in pictograms). Shaded areas indicate standard error. **(B)** Peak extension and flexion angles. Differently colored bars represent the different exoskeleton configurations. Error bars indicate standard error. Symbols next to brackets indicate significant differences between exoskeleton configurations. ^*^p ≤ 0.05. ^**^p ≤ 0.01.

Exoskeleton configuration had significant effects on maximum plantarflexion (*df*_cond._ = 3, *df*_err._ = 24, *F* = 44.481, *p* < 0.001, η^2^ = 0.848), maximum dorsiflexion (*df*_cond._ = 3, *df*_err._ = 24, *F* = 12.089, *p* < 0.001, η^2^ = 0.602), and maximum knee extension (*df*_cond._ = 3, *df*_err._ = 24, *F* = 3.709, *p* = 0.025, η^2^ = 0.317) (Figure 6).

Maximum plantarflexion in the *mono-articular no-spring condition* was higher than those in all other *exoskeleton conditions* (*df* = 8, all *T* ≥ 7.594, all *p* < 0.001, all *d* ≥ 2.531). Maximum plantarflexion in the *bi-articular plus spring condition* was higher than that in the *powered-off condition* (*df* = 8, *T* = 2.949, *p* = 0.019, *d* = 0.983). In all *active exoskeleton conditions*, the maximum dorsiflexion angle before push-off was lower than that in the *powered-off condition* (*df* = 8, all *T* ≥ 3.720, all *p* < 0.006, all *d* ≥ 1.240).

Maximum knee extension (just before push-off) in the *mono-articular no-spring condition* was higher than those in all the other exoskeleton conditions (*df* = 8, all *T* ≥ 2.583, all *p* < 0.033, all *d* ≥ 0.861). We found no significant effects of exoskeleton configuration on hip joint angle or step length.

**Figure 7:**
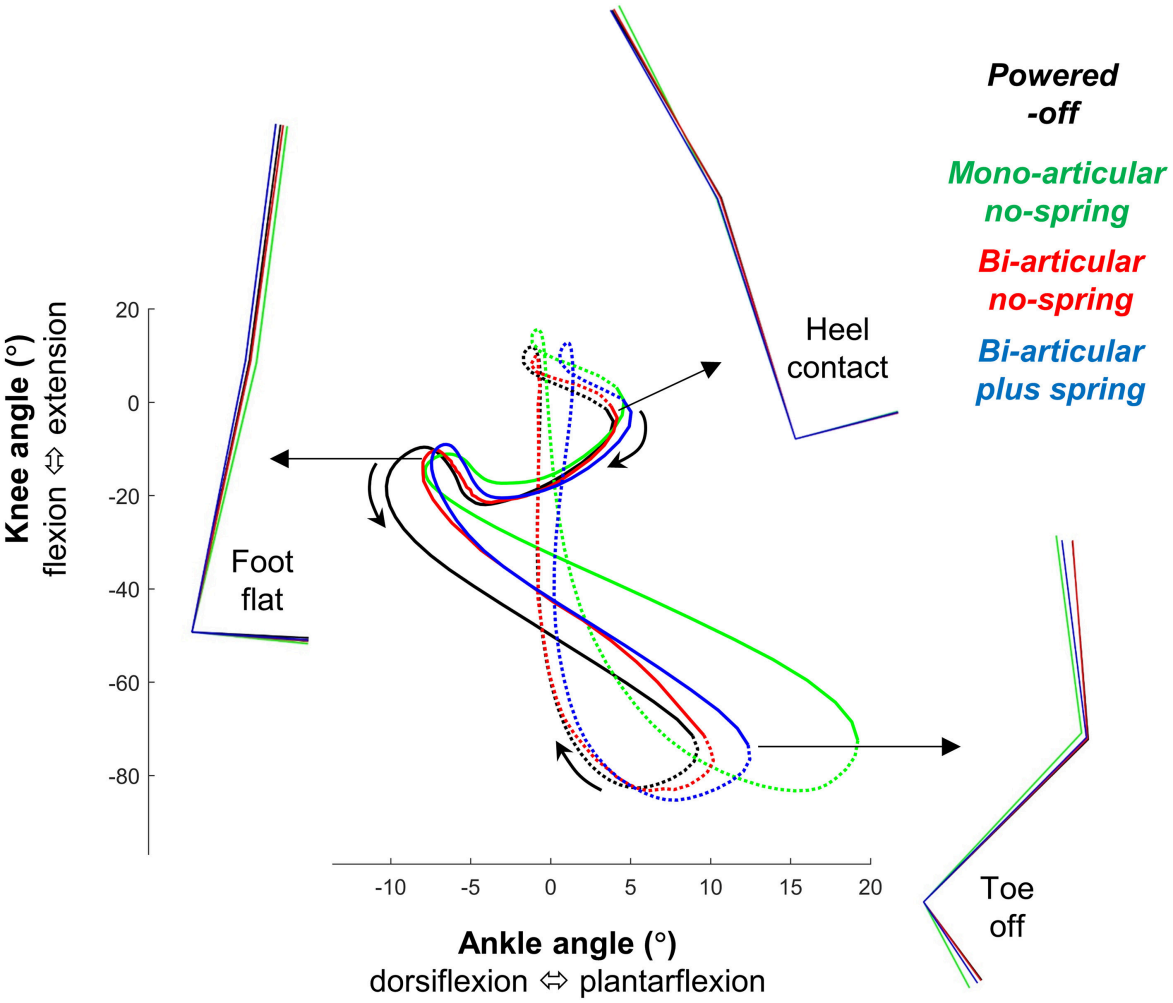
Bi-articular coupling. Plot shows knee angle plotted on the vertical axis vs. ankle angle on the horizontal axis as in Robertson et al. (2004). Full lines indicate stance phase. Dotted lines indicate swing phase. Colored lines indicate the population average of different conditions. Stick figures indicate lower limb kinematics position at different points on the gait cycle. This figure shows that in the *bi-articular conditions* the coupling between the knee and ankle angle is more similar to the *powered-off condition*.

Overall, in the *bi-articular conditions* the coupling between the knee and ankle angle was more similar to the *powered-off* condition than in the *mono-articular condition* (Figure 7).

## Discussion

Our aim was to evaluate an exoskeleton with a bi-articular actuation configuration that mimicked the eccentric and concentric behavior of the m. gastrocnemius and to compare this condition with one that mimicked only the concentric behavior of the m. gastrocnemius and one that mimicked the concentric behavior of the m. soleus. We found that the *bi-articular plus spring condition* provided the highest reduction in metabolic cost (13% compared to p*owered-off*, Figure 2). The *bi-articular no-spring condition* and the *mono-articular no-spring condition* both provided reductions of ~6% compared to *powered-off*. In contrast to our hypothesis we did not find the highest reduction in m. soleus EMG in the *mono-articular condition*. On average m. gastrocnemius EMG appeared to be lower in the *biarticular conditions* than in the *mono-articular condition* but this was not significant.

Peak exoskeleton ankle moment and exoskeleton ankle work rate values in the *active* conditions were respectively around 17 and 23% of the biological moment and work rate values reported in Winter (1983). In the *bi-articular* conditions there was an exoskeleton knee flexion moment and positive exoskeleton knee flexion work during the phase when the biological knee moment is in the extension direction and the biological knee work is negative (Winter, 1983) so the knee action of the exoskeleton was different than the action of the net of all the biological knee muscles. However, it is known that non-biological exoskeleton behavior can sometimes be more effective than biological behavior (Mooney and Herr, 2016; Uchida et al., 2016).

The reduction in the m. soleus EMG in the *mono-articular no-spring condition* is consistent with results from other studies showing that mono-articular ankle exoskeletons can reduce the m. soleus EMG (Sawicki and Ferris, 2008; Galle et al., 2013, 2017). The fact that the reduction in the m. soleus EMG is not the highest in the *mono-articular no-spring condition* despite the fact that in this condition the assistance is in parallel with the m. soleus may have been due to the higher maximum plantarflexion in the *mono-articular no-spring condition* (Figure 6). It appears that the participants utilized the exoskeleton assistance in that condition to increase plantarflexion instead of maximizing reductions in the m. soleus EMG while keeping kinematics invariant. Similar kinematic changes have also been observed in other studies with ankle exoskeletons and exosuits (Koller et al., 2015; Mooney and Herr, 2016; Quinlivan et al., 2017). The absence of a similarly large increase in plantarflexion in the *bi-articular conditions* may have been because the attachment points of the pneumatic muscle came closer to each other when the knee started to flex during push-off. Overall it appears that the *bi-articular conditions* allowed for more natural knee and ankle kinematics than the *mono-articular condition* (Figure 7). For future exoskeleton designs it appears that this type of bi-articular configuration could be useful if the objective is to assist while maintaining kinematics that are as close as possible to natural biological kinematics.

Though we did not find increases in knee flexion in the *bi-articular conditions*, we did find reductions in the biceps femoris EMG at the end of swing. Because the biceps femoris is in part a knee flexor muscle it could be that the higher reduction in biceps femoris EMG is because the *bi-articular conditions* effectively assisted the knee flexion function. Although it seems logical that we did not find these reductions in the m. biceps femoris EMG in the *mono-articular condition*, we know from other studies that the m. biceps femoris EMG can actually also be reduced with a mono-articular exoskeleton, for example by providing higher assistive moments (Galle et al., 2017).

The additional assistance with knee flexion may have contributed to our finding of the highest reduction in metabolic rate in the *bi-articular plus spring condition*. We found that the sum of the positive ankle and knee work from the exoskeleton had the strongest correlation with the metabolic rate reduction (Figure 4). It appears that this work sum was the highest in the *bi-articular plus spring condition* due in part to the additional work delivered at the knee. Another explanation is that the *bi-articular plus spring condition* provided the highest negative work assistance at the ankle thanks to the early actuation time selected by the participants and the elastic element (Figure 3). Following the same hypothesis that was suggested in a study with an elastic ankle exoskeleton (Collins et al., 2015) the additional assistance during the isometric contraction phase of the biological plantarflexors could explain the higher metabolic reduction in the *bi-articular plus spring condition*.

The participants preferred earlier actuation onset timing in the *bi-articular plus spring condition* than in the *mono-articular no-spring condition*. It has been suggested that due to attributes such as low weight and elastic behavior, pneumatic muscles are useful for applications involving human interaction, such as exoskeletons (Daerden and Lefeber, 2002). However, it is also known that the contraction forces of pneumatic muscles are highest when they are elongated, and that pneumatic muscles can cause the ankle to plantarflex earlier than normal (Gordon et al., 2006). Furthermore, a simulation study of walking with an elastic ankle-foot orthosis showed that a soleus-mimicking mono-articular orthosis could cause unnatural premature knee extension during midstance (Arch et al., 2016). This inelastic pneumatic muscle behavior at maximum elongation combined with the potential to cause unnatural knee extension when actuation would be too early may explain why the participants preferred a later actuation onset timing in the *mono-articular no-spring condition*.

A limitation of our study is that the timing for each condition was selected based on perception tests. The perceived optimal timing in the *mono-articular no-spring condition* was not significantly different from the optimal timing found in previous studies (Malcolm et al., 2013; Galle et al., 2017), which suggests that the participants were relatively good at identifying their optimal timing. However, we do not have direct evidence that the participants correctly selected the optimal timing in the *bi-articular conditions*. Another limitation is that the actuator configuration was not the only difference between the conditions; there were also differences in other parameters such as timing, peak moment, work, etc. It is impossible to vary one parameter in isolation and keep every other actuation parameter constant because changes in exoskeleton parameters usually also change the kinematics (Galle et al., 2015; Koller et al., 2015; Mooney and Herr, 2016; Quinlivan et al., 2017). This challenge seems to be common in the field, and as far as we know, there are only a very small number of experimental studies that describe within-subject comparisons of exoskeleton conditions (Ding et al., 2017). In an ideal case, either a constant rate of work should be delivered in all the conditions or all the parameters of the entire actuation profile should be optimized for each condition. Delivering a constant rate of work in all conditions would allow to answer the question which is the best configuration to deliver a certain rate of work. In the current experiment it could be that the *bi-articular plus spring* provided the highest metabolic cost reduction simply because this allowed to provide more mechanical work assistance to the ankle plus the knee. However, recent studies learn that exoskeleton mechanical work only is not necessarily related to reduction in metabolic cost (Jackson and Collins, 2015; Zhang et al., 2017). Optimizing the entire actuation profile in order to identify the best profile for each configuration would require using human-in-the-loop optimization (Zhang et al., 2017). However, this approach was not feasible at the time of the data collection in our study. Another limitation in the interpretation of the results is that we only calculated the total power from the pneumatic muscle (and the series elastic cord and/or non-elastic cord in the *bi-articular conditions*). Calculating the power from each component separately would allow to discuss how each component contributes to the power delivered by the exoskeleton (Eslamy et al., 2015; Yandell et al., 2017). An alternative approach to conduct our study could have been to use two single joint actuators: one at the ankle and one at the knee. By using two separate actuators it would be possible to separate the assistive effects at the ankle and the knee. The two actuators could be programmed to behave as if there is a biarticular connection from the foot to the thigh or even different combinations of ankle and knee actuation could be tested. The fact that the actuation profiles were not fully optimized probably contributed to the fact that none of the exoskeleton conditions reduced the metabolic cost below the level of walking without an exoskeleton.

Another reason why we did not find reductions in metabolic cost below that of walking without an exoskeleton may have been the additional mass of the thigh segments of the exoskeleton. While our exoskeleton weighed relatively less than other knee-ankle-foot exoskeletons such as the ones from Chen et al. (2016) (3.5 kg for one side) and Sawicki and Ferris (2009) (2.9 kg for one side) it still weighed a considerable total of 1.9 kg per side. Based on a literature regression equation (Browning et al., 2007), we estimated that the weight of the thigh segments would have caused a penalty in metabolic cost of 6.7%. The metal bars on both the medial and lateral side of the legs might have encumbered walking and caused participants to take wider steps. Some designs from other exoskeletons that use struts only on the lateral side of the leg might solve this problem (Suzuki et al., 2007; Esquenazi et al., 2012). Our exoskeleton design used a simple hinge joint for the knee, whereas the knee actually has a moving axis of rotation (Witte et al., 2017). The use of design principles from soft exosuits for the knee (Wehner et al., 2013; Park et al., 2014) could potentially both help reduce the weight of our device and resolve problems with joint axis alignment.

For clinical application of our exoskeleton the design and actuation profiles would need to be optimized. For example, the optimal timing could be different for every single patient as was found in stroke patients in Awad et al. (2017). It is unknown if the biarticular configuration and spring would have benefits in different populations such as patients since the current study was only conducted in a small sample of nine healthy volunteers and at a higher walking speed than patients typically use.

In conclusion, we found that a bi-articular exoskeleton configuration that mimics the m. gastrocnemius can reduce the metabolic cost of walking and reduce biceps femoris EMG. The following factors could have contributed to the higher reduction in metabolic rate in the *bi-articular plus spring condition*: closer to normal ankle and knee kinematics, additional assistance with knee flexion and higher total mechanical work assistance. However, we do not know to what extent each of these factors contributed to the metabolic cost result. Future exoskeleton designs could leverage each of these factors, possibly with different exoskeleton designs than ours, for example with separate ankle and knee actuation or with more lightweight and soft structures. Knowledge about specific effects of exoskeleton configuration on metabolic costs and muscle activation could be applied to providing customized assistance for different gait impairments or injuries and could also lead to novel experiments aimed at investigating the separate roles of the m. gastrocnemius and soleus.

## Author contributions

PM, SG, WD, and DD designed the study. PM and SG performed data collection. PM performed the data analysis and drafted the manuscript. All authors edited and revised the analyses and the manuscript and gave final approval for publication.

### Conflict of interest statement

The authors declare that the research was conducted in the absence of any commercial or financial relationships that could be construed as a potential conflict of interest.

## Supplementary material

Movie 1 Participant walking in different exoskeleton configurations. Video shows the *bi-articular plus spring condition, bi-articular no-spring condition, mono-articular no-spring condition, powered-off condition*, and *no-exoskeleton condition*.

Data Sheet 1 Study dataset containing results for metabolic cost, exoskeleton kinetics, EMG and kinematics. The data is saved in .mat-format and can be opened with MATLAB. Data is organized similar to exoskeleton datasets from Jackson and Collins (2015). Timeseries variables are stored as three-dimensional matrices where the first dimension is stridetime, the second dimension is participant number and the third dimension is condition number. Metric variables are stored as two-dimensional matrices where the first dimension is participant number and the second dimension is condition number. The conditions are numbered as follows: 1 = *bi-articular plus spring condition*, 2 = *bi-articular no-spring condition*, 3 = *mono-articular no-spring condition*, 4 = *powered-off condition*, 5 = *no-exoskeleton condition*.
